# Dr. J. P. Barret

**Published:** 1860-01

**Authors:** 


### NECROLOGICAL NOTICES.

DIED, recently, at Abbeville, South Carolina, Dr. J. P. BARRET, an eminent practitioner, and a gentleman of great moral worth. His disease was cancer of the stomach, under which he had suffered long and severely. He was a valued contributor to our periodical literature, especially to the *Charleston Medical and Surgical Journal*, and was at one time President of the South Carolina State Medical Association.

